# Combined ginger extract & Gelam honey modulate Ras/ERK and PI3K/AKT pathway genes in colon cancer HT29 cells

**DOI:** 10.1186/s12937-015-0015-2

**Published:** 2015-04-01

**Authors:** Analhuda Abdullah Tahir, Nur Fathiah Abdul Sani, Noor Azian Murad, Suzana Makpol, Wan Zurinah Wan Ngah, Yasmin Anum Mohd Yusof

**Affiliations:** 1 Department of Biochemistry, Faculty of Medicine, Universiti Kebangsaan Malaysia, Jalan Yaacob Latif, Bandar Tun Razak, Cheras, 56000 Kuala Lumpur, Malaysia; 2 Centre of Lipid Engineering and Applied Research (CLEAR), Universiti Teknologi Malaysia, Jalan Semarak, 50480 Kuala Lumpur, Malaysia

**Keywords:** Ginger, Gelam honey, HT29 colon cancer cells, Ras/ERK and PI3K/AKT pathways

## Abstract

**Background:**

The interconnected Ras/ERK and PI3K/AKT pathways play a central role in colorectal tumorigenesis, and they are targets for elucidating mechanisms involved in attempts to induce colon cancer cell death. Both ginger (*Zingiber officinale*) and honey have been shown to exhibit anti-tumor and anti-inflammation properties against many types of cancer, including colorectal cancer. However, there are currently no reports showing the combined effect of these two dietary compounds in cancer growth inhibition. The aim of this study was to evaluate the synergistic effect of crude ginger extract and Gelam honey in combination as potential cancer chemopreventive agents against the colorectal cancer cell line HT29.

**Methods:**

The cells were divided into 4 groups: the first group represents HT29 cells without treatment, the second and third groups were cells treated singly with either ginger or Gelam honey, respectively, and the last group represents cells treated with ginger and Gelam honey combined.

**Results:**

The results of MTS assay showed that the IC_50_ of ginger and Gelam honey alone were 5.2 mg/ml and 80 mg/ml, respectively, whereas the IC_50_ of the combination treatment was 3 mg/ml of ginger plus 27 mg/ml of Gelam honey with a combination index of < 1, suggesting synergism. Cell death in response to the combined ginger and Gelam honey treatment was associated with the stimulation of early apoptosis (upregulation of *caspase 9* and *IκB* genes) accompanied by downregulation of the *KRAS*, *ERK*, *AKT*, *Bcl*-*xL*, *NFkB* (*p65*) genes in a synergistic manner.

**Conclusions:**

In conclusion, the combination of ginger and Gelam honey may be an effective chemopreventive and therapeutic strategy for inducing the death of colon cancer cells.

## Background

Colorectal cancer (CRC) is the third most common cancer and the second leading cause of death among cancers worldwide [1]. Malaysia has also experienced the same increasing trend of colorectal cancer, the second most common cancer in both males and females [2]. Colorectal carcinogenesis is a multistep process that transforms colonic epithelial cells into colon adenocarcinoma cells involving mutation of the *APC* gene as an initiator followed by mutations of the genes *KRAS*, *PIK3CA*, transforming growth factor-β and *p53* [3,4].

KRAS protein plays a central role in controlling the activity of several crucial downstream signaling pathways such as P13K/AKT/mTOR and Ras/Raf/ERK that regulate normal cellular proliferation, differentiation and survival [5]. Mutations of the *KRAS* proto-oncogene and any of its downstream elements will lead to abnormal signaling and activation of these pathways, resulting in uncontrolled cell growth [6]. Mutations of the kras proto-oncogene have been found in 30% to 50% of early adenoma to intermediate adenoma of colon cancer [6]. Activation of the PI3K/AKT pathway has also been shown to activate NFκB through phosphorylation of IKK, which in turn phosphorylates IκBα and RELA/p65 [7]. The Ras/ERK, PI3K/AKT and NFκB pathways are interconnected and play a central role in colorectal tumorigenesis [6,7]. Thus, targeting these crosstalk pathways using combined strong chemopreventive agents may lead to greater inhibition of colorectal carcinogenesis.

Natural dietary phytochemicals have potential to be used in cancer therapy because in addition to reducing adverse side effects, they also improve the effectiveness of chemotherapeutics [8]. These compounds may interfere with carcinogenic processes at various levels, both by blocking initiation and by suppressing the later stages involving promotion, progression, angiogenesis, invasion and metastasis [9]. A growing number of *in vitro* and *in vivo* studies have suggested that combinations of dietary phytochemical may be far more effective at protecting against cancer compared to individual compounds [9-12]. Combination treatment with resveratrol and black tea at low dose synergistically inhibits the growth of skin cancer through induction of apoptosis and inhibition of MAPK signaling [10], and the combination of curcumin with garcinol induces apoptosis through increased activity of caspases 3 and 9 in pancreatic cancer cells (BxPC-3 PaCa and PANC-1) [13]. The combination of *zingiber officinale* and *piper retrofractum* displays increased apoptosis activity in WiDr myeloma cells [14].

Ginger (*Zingiber officinale*) contains phenolic compounds such as gingerol, shogaol, paradol, and zerumbone that have been demonstrated to have antioxidant, anti-tumor and anti-inflammatory properties [15,16]. Ginger extract and its bioactive compounds were shown to inhibit the growth of oral cancer [16], colon cancer [17], gastric cancer, [18], skin cancer [19], liver cancer [20,21], and ovarian cancer [22] cells mainly by inhibiting proliferation, inducing apoptosis and inactivating NFkB activity. Ginger supplementation at 2 g daily for 28 days to 20 patients at high risk of colorectal cancer resulted in a reduced proliferation rate (reduced expression of Ki-67) but had less effect on apoptosis in the crypts of normal appearing colonic mucosa cells [23].

Gelam and Tualang honeys from Malaysia exhibit anti-cancer properties such as inhibition of cell proliferation and DNA damage, induction of apoptosis and cell cycle arrest of liver and colon cancer cells [24,25], human osteosarcoma cells (HOS) [26], breast cancer cells (MCF-7 and MDA-MB-231) and cervical cancer cells (HeLa) [27]. Gelam honey also exhibits anti-inflammatory effects by significantly decreasing the production of pro-inflammatory cytokines such as NO, PGE2, TNF-α, and IL-6 in carrageenan-induced acute paw edema in rats [28]. The anti-inflammatory and anti-tumor properties of Gelam honey were suggested to be due to the high content of phenolic compounds such as gallic acid, chlorogenic acid, caffeic acid, *p*-coumaric acid, ferulic acid, ellagic acid, quercetin, hesperetin and chrysin [24,28].

Our earlier study showed that the combination of ginger and Gelam honey enhances the chemotherapeutic effect of 5-fluorouracil against colon cancer line HTC116 [29]. However, the mechanisms involved are still unclear. In the present study, crude extracts of ginger and Gelam honey were used in combination to determine their potential synergistic chemopreventive effect against HT29 colorectal cancer cells, focusing on molecular mechanisms involving the Ras/ERK and PI3K/AKT pathways that may represent the early stage of colorectal cancer formation.

## Methods

### Cell culture

Human colorectal adenocarcinoma cell line HT29 was obtained from the American Type Culture Collection (ATCC, Rockville, MD, USA). HT29 cells were chosen because they are sensitive to treatment with chemotherapeutic drugs and maintain as a nonpolarized, undifferentiated multilayer. The cell line was maintained in a T-25 flask containing McCoy’s 5A Medium Modified with L-glutamine (Gibco Life Technologies, Inc., Grand Island, NY, USA) and supplemented with 10% fetal calf serum (FCS), 100 U/mL penicillin-streptomycin (Gibco Life Technologies, Inc., Grand Island, NY, USA) and 100 U/mL amphotericin B (PAA Laboratories GmbH, Pasching, Austria) at 37°C in a humidified atmosphere of 5% CO_2_. The cell line was maintained as a monolayer and split regularly before reaching 80-90% confluence.

### Source of ginger extract and Gelam honey

The crude ginger extract was supplied by Dr. Noor Azian Murad of the Centre of Lipid Engineering and Applied Research (CLEAR), Universiti Teknologi Malaysia. Ginger extract was isolated by ultrasonic assisted extraction (UAE) using water at 20–2200 kHz frequency for 10–120 minutes and temperature of 30-140°C. The extract was filtered into a 20 ml falcon tube and stored at −80°C. The frozen extract was freeze-dried to form a powder and stored at room temperature before use. Local fresh Malaysian monofloral Gelam honey was provided by the National Apiary, Department of Agriculture, Batu Pahat, Johor, Malaysia. The Gelam honey was packaged in plastic containers and sent to SINAGAMA, Malaysian Nuclear Agency for sterilization by gamma irradiation. The irradiation process was performed at 25 kGy and the honey was subsequently stored at 4°C away from direct light for future use. The ginger and Gelam honey were diluted with complete culture medium at varying concentrations for the experiments.

### Cell viability assay

Cell viability was assessed by using MTS (3-(4,5-dimethylthiazol-2-yl)-5-(3-carboxymethoxyphenyl)-2-(4-sulphenyl)-2H-tetrazolium) (Promega, Madison, WI, USA). HT29 cells were plated in 96-well plates at a density of 2 × 10^4^ cells per 100 μL complete medium (McCoy 5A Medium Modified) and incubated for 24 hours at 37°C in a 5% CO_2_ incubator. After 24 hours of incubation, the culture medium was removed and replaced with 100 μL of new complete medium for the control group, 100 μL of ginger (2–10 mg/ml), 100 μL of Gelam honey (20–100 mg/ml) or the combination of both ginger and Gelam honey at various concentrations to the appropriate wells. Three replicates were performed for each treatment. The plate was then wrapped in aluminum foil and subsequently re-incubated for another 24 hours. After the incubation period, the culture medium was discarded and replaced with MTS solution (20 μL MTS diluted in 100 μL complete medium) in each well before being incubated again at 37°C, 5% CO_2_ for another 2 hours. The resulting products were determined spectrophotometrically at an absorbance of 490 ηm using VERSAmax microplate reader (Molecular Devices, Sunnyvale, CA, USA). The results are expressed as a percentage for each treatment group relative to the control in the absence of ginger and Gelam honey.

### Treatment of HT29 cells with the combination of ginger and Gelam honey

HT29 cells were incubated for 24 hours with a fixed concentration of 2, 3 or 4 mg/ml of ginger and varying concentrations of Gelam honey (1–70 mg/mL). The combination concentrations that were used were lower than the IC_50_ of single treatment with ginger and Gelam honey. The data were then analyzed using the Chou and Talalay formula

$$ CI=\left(dA/DA\right)+\left( dB/DB\right) $$

where dA = the IC_50_ of compound A in combination, DA = the IC_50_ of single compound A, dB = IC_50_ of compound B in combination, DB = the IC_50_ of single compound B. Additivity, CI = 1; antagonism, CI = >1; synergism, CI = <1.

### Cell death detection by ELISA assay

The mechanism of cell death was quantitatively determined using the Cell Death Detection ELISA PLUS 96 kit (Roche Applied Science, Mannhein, Germany) according to the manufacturer’s protocol. Briefly, 2 × 10^4^ HT29 cells were seeded in 96-well plates and were incubated overnight at 37°C in a 5% CO_2_ incubator. Following an overnight incubation period, the cells were treated with either ginger, Gelam honey or the combination for another 24 hours. The treated cells were lysed with 200 ml of lysis buffer and incubated for 30 minutes at room temperature. The lysed solution (20 μL) was placed in triplicate into wells of a streptavidin-coated microplate followed by the addition of 80 μL of immunoreagent containing a mixture of anti–histone-biotin and anti–DNA-POD. The plate was covered with an adhesive foil cover and incubated for 2 hours at 25°C in a shaking incubator at 300 rpm. The unbound antibody was washed three times with 250–300 ml incubation buffer. ABTS (2,2’-azinobis-3-ethyl-benzothiazoline-6-sulfonic acid) solution (100 μL) was added to each well and incubated on a plate shaker for 10–20 minutes. The amount of nucleosomes retained by the POD in the immunocomplex, corresponding to the extent of apoptosis, was quantitatively determined photometrically with ABTS solution as substrate using a microplate reader at a wavelength of 405 nm and reference wavelength of 490 nm.

### Real-Time Polymerase Chain Reaction (RT-qPCR)

A total of 1 × 10^6^ HT29 cells were plated in a 60 mm culture dish and incubated for 24 hours at 37°C in 5% CO_2_. The cells were then treated with different doses of ginger, Gelam honey or the combination of ginger and Gelam honey for 24 hours. The cells were harvested, cellular RNA was extracted using an RNeasy Mini kit (QIAGEN, Valencia, CA, USA) in an RNase-free environment according to the manufacturer’s instructions, and it was subsequently purified by ethanol precipitation. RNA concentration and purity were determined based on measurement of the absorbance at 260 nm and 280 nm. For each sample, approximately 200 ng of RNA was used to synthesize cDNA. RNA was initially mixed with ultra-pure distilled water up to 16 μl and mixed with 4 μl of iScript™ Reverse Transcription Supermix (BIO-RAD, Hercules, CA, USA), which contains reverse transcriptase, RNase inhibitor, dNTPs, primer, MgCl_2_ and stabilizers. PCR-reaction primers were designed using the National Centre for Biotechnology Information (NCBI) website. The primer sequences that were used are shown in Table 1.

**Table 1:** **Primer sequences for quantitative real-time RT-PCR**

Gene	Forward primer (F)	Reverse primer (R)
**GADPH**	TCCCTGAGCTGAACGGGAAG	GGAGGAGTGGGTGTCGCTGT
**Kras**	TAGTTGGAGCTGGTGGCGTA	CCTCTTGACCTGCTGTGTCG
**Erk**	ACAGGCTGTTCCCAAATGCT	CGAACTTGAATGGTGCTTCG
**Akt**	GTCGCCTGCCCTTCTACAAC	CACACGATACCGGCAAAGAA
**Bcl-xl**	GCATTGTGGCCTTTTTCTCC	GCTGCTGCATTGTTCCCATA
**Caspase 9**	GAGGGAAGCCCAAGCTCTTT	CACTGGGTGTGGGCAAACTA
**P65**	CCTGGGAATCCAGTGTGTGA	GCACTGTCACCTGGAAGCAG
**IκBα**	GAAGTGATCCGCCAGGTGAA	CTCACAGGCAAGGTGTAGGG

RT-qPCR was performed using SYBR-green detection (BIO-RAD, Hercules, CA, USA) in an iQ5 Real-time cycler machine (BIO-RAD, Hercules, CA, USA). The cycling conditions were as follows: initial denaturation at 95°C for 3 min and amplification for 40 cycles (95°C for 10 sec for the denaturation, 56°C for 30 sec for the annealing and extension). The quality of the PCR products was checked by 2% agarose gel electrophoresis. In all cases, single bands of the expected size were observed. The specificity of each PCR product was further assessed by melting curve analysis. The relative amount of gene expression normalized to the internal control GAPDH

$$ \mathrm{Relative}\ \mathrm{Expression}\ \mathrm{Value}\ \left(\mathrm{REV}\right) = {2}^{\left(\mathrm{C}\mathrm{t}\ \mathrm{GAPDH}-\mathrm{C}\mathrm{t}\ \mathrm{t}\mathrm{arget}\ \mathrm{gene}\right)} $$

where Ct = the cycle number at threshold level.

### Statistical analysis

All data were statistically analyzed using Statistical Package for Social Sciences (SPSS) software version 20. The normality of each test was assessed using the Shapiro-Wilk test. Normally distributed data were then tested with one-way ANOVA followed by Tukey's HSD test as a post-hoc test. For data values that were not normally distributed, they were analyzed using non-parameter tests (Kruskal-Wallis and Mann–Whitney). All data are presented as the mean ± standard error (SEM), and p < 0.05 was considered statistically significant.

## Results

### Effects of treatment with ginger and Gelam honey on the viability HT29 cell line

**Figure 1:**
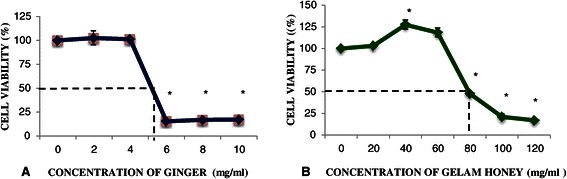
**Effects of ginger and Gelam honey alone on viability of HT29 cells.** Viability of HT29 cells with treatment of Ginger **(A)** and Gelam honey **(B)** was determined by MTS assay after 24 hour treatment with increasing concentrations of ginger or Gelam honey. Data are presented as the mean ± SEM from three independent experiments. *(p < 0.05) significant compared to without treatment.

Figure 1A and B show the IC_50_ of ginger extract and Gelam honey to be 5.2 mg/ml and 80 mg/ml, respectively. The viability of HT29 cells did not show any changes when treated at low concentrations with ginger (2 and 4 mg/ml) or Gelam honey (20, 40 and 60 mg/ml). Whereas at high concentration, both ginger and Gelam honey decreased the viability of HT29 cells significantly (p<0.05) compared to untreated cells.

### Effects of combined treatment with ginger and Gelam honey on the viability HT29 cell line

**Figure 2:**
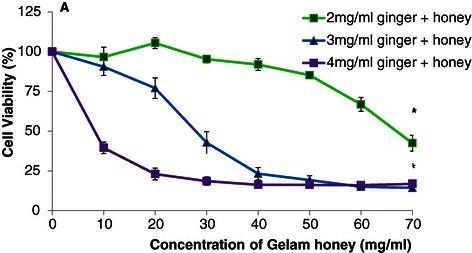
**Effects of ginger and Gelam honey combined on viability of HT29 cells.** Cell viability was determined by MTS assay after 24 hour treatment with increasing concentrations of ginger and Gelam honey combined. Data are presented as the mean ± SEM from three independent experiments. *(p < 0.05 ) significant compared to without treatment.

The combined treatment doses chosen for ginger (2, 3 and 4 mg/ml) and Gelam honey (10 to 70 mg/ml) were lower than the IC_50_ values of the individual treatments. A dose of 2 mg/ml of ginger needed a higher concentration of honey (67 mg/ml) to achieve the required IC_50_, whereas both 3 and 4 mg/ml of ginger required lower concentrations of Gelam honey (27 mg/ml and 10 mg/ml, respectively) to achieve the required IC_50_ (Figure 2). Table 2 shows that the combination index (CI) for 2 mg/ml of ginger with 67 mg/ml of Gelam honey is 1.21 (CI > 1), which suggests an antagonistic effect. The combinations of 3 and 4 mg/ml of ginger with 27 and 10 mg/ml of Gelam honey showed CI values of 0.92 and 0.90, respectively, indicating a synergistic effect between the two compounds.

**Table 2:** **The combination index (CI) for combined ginger and Gelam honey on HT29 cell line**

Ginger (mg/ml)	IC _ 50 _ Gelam honey (mg/ml)	Combination Index (CI)
**2**	67	1.21
**3**	27	0.92
**4**	10	0.90

Combination index, CI was determined according to Chou and Talalay [30] as explained in the Methods section. Additivity, CI = 1; antagonism, CI = >1; synergism, CI = <1.

### Effects of single and combined treatments with ginger & Gelam Honey on apoptosis of HT29 cells

**Figure 3:**
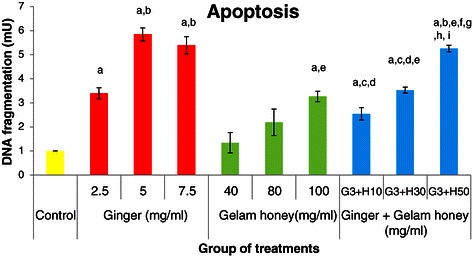
**Effects of ginger and Gelam honey on apoptosis of HT29 cells.** The apoptotic effects of single and combined treatments with ginger and Gelam honey were determined by Cell Death Detection ELISA after 24 hours. Data are presented as the mean ± SEM from three independent experiments. ^a^(p < 0.05) significant compared to control, ^b^(p < 0.05) significant compared to 2.5 mg/ml of ginger, ^c^(p < 0.05) significant compared to 5.0 mg/ml of ginger, ^d^(p < 0.05) significant compared to 7.5 mg/ml of ginger, e(p < 0.05) significant compared to 40 mg/ml of Gelam honey, ^f^(p < 0.05) significant compared to 80 mg/ml of Gelam honey, ^g^(p < 0.05) significant compared to 100 mg/ml of Gelam honey, ^h^(p < 0.05) significant compared to combination of 3 mg/ml ginger and 10 mg/ml Gelam honey, ^i^(p < 0.05) significant compared to combination of 3 mg/ml ginger and 30 mg/ml Gelam honey.

As illustrated in Figure 3, treatment with ginger elicited a dose-dependent increase in DNA fragmentation that was higher compared to treatment with honey, indicating a higher rate of apoptosis. Interestingly, when 3 mg/ml of ginger was combined with 50 mg/ml of honey, a synergistic increase in the rate of apoptosis was seen compared to treatment with honey alone.

### Effect of ginger & Gelam honey alone and in combination on genes in the Ras/ERK & PI3k/AKT pathways

**Figure 4:**
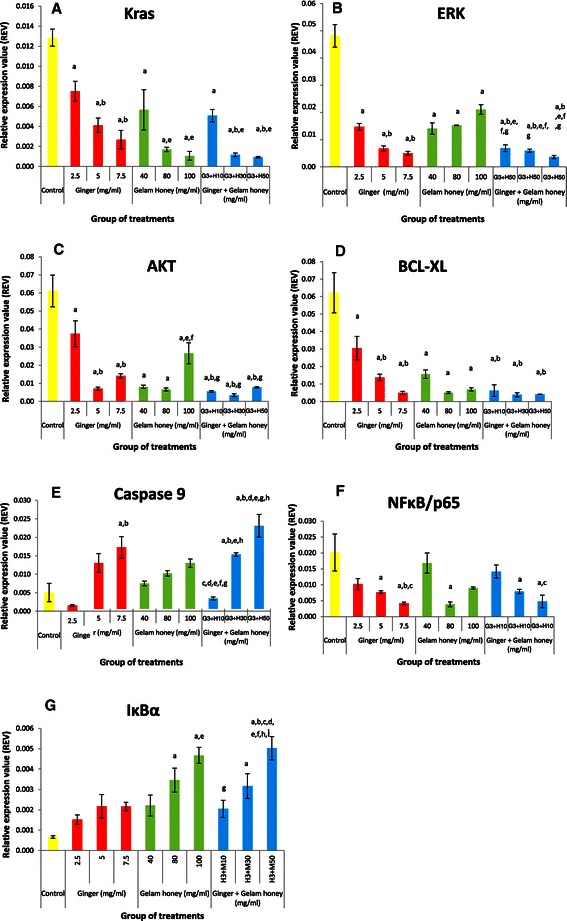
**Effects of single and combined treatments with ginger & Gelam honey on expression of Ras**/**ERK & PI3K**/**AKT pathway genes in HT29 cells.** RNA was isolated, reverse transcribed to cDNA, and then amplified by a real-time PCR detection system to measure mRNA levels of *GAPDH*, *KRAS***(A)**, *ERK***(B)***AKT***(C)**, *Bcl*-*xL***(D)**, *caspase 9***(E)**, *NFĸB/p65***(F)**, *IĸBα***(G)**. Target genes were normalized to *GAPDH*. The data are presented as the mean ± SEM from three independent experiments. ^a^(p < 0.05) significant compared to control, ^b^(p < 0.05) significant compared to 2.5 mg/ml of ginger, ^c^(p < 0.05) significant compared to 5.0 mg/ml of ginger, ^d^(p < 0.05) significant compared to 7.5 mg/ml of ginger, e(p < 0.05) significant compared to 40 mg/ml of Gelam honey, ^f^(p < 0.05) significant compared to 80 mg/ml of Gelam honey, ^g^(p < 0.05) significant compared to 100 mg/ml of Gelam honey, ^h^(p < 0.05) significant compared to combination of 3 mg/ml ginger and 10 mg/ml Gelam honey, ^i^(p < 0.05) significant compared to combination of 3 mg/ml ginger and 30 mg/ml Gelam honey.

Modulation of the gene expression of *KRAS*, *ERK*, *AKT*, *Bcl*-*xL*, *caspase 9*, *p65* and *IκBα* involved in the Ras/ERK and PI3K/AKT pathways was investigated with ginger and Gelam honey. The gene expression levels of *KRAS*, *ERK* and *AKT* involved in the Ras/ERK/PI3K/AKT pathways were high in control cells (Figure 4A,B, and C). Treatment with ginger or Gelam honey alone resulted in the downregulation of the *KRAS* gene compared to control. However, a synergistic downregulation of *KRAS* was observed with combination treatment, especially at the dose of 3 mg/ml ginger in combination with either 30 or 50 mg/ml honey. As shown in Figure 4B, treatment with ginger alone downregulated expression of the *ERK* gene in a dose dependent manner, but combination of ginger with Gelam honey showed a greater effect on downregulating *ERK* gene expression compared to ginger or Gelam honey treatment alone. Treatment with either ginger or Gelam honey was found to downregulate *AKT* gene expression independently of the dose (Figure 4C). However, combination of both ginger and Gelam honey yielded higher downregulation of *AKT* gene expression at a much lower concentration compared to either treatment alone. The expression of *Bcl*-*xL*, an anti-apoptosis gene that was upregulated in the control group, was downregulated by either individual treatment, whereas combination treatment showed an even greater effect (Figure 4D). Conversely, expression of the pro-apoptosis gene *caspase 9* (Figure 4E), which was downregulated in the untreated control group, was upregulated in treatments with high concentrations of ginger or Gelam honey alone, and a synergistic upregulation of *caspase 9* was observed with the combined treatment at a much lower dose. As seen in Figure 4F, the inflammatory gene *NFκB* (*p65*), which is upregulated in cancer cells, was downregulated in treatments with ginger and Gelam honey alone or in combination, whereas the inhibitory gene *IκBα* (Figure 4G), which is downregulated in cancer cell lines, was highly upregulated with the combination treatment.

## Discussion

Chemoprevention, the use of natural dietary compounds to block, inhibit, and reverse the process of tumor formation, is a relatively new strategy to prevent cancer. Many studies have focused on natural products such as vegetables, fruits, spices, teas, herbs and phytochemical constituents such as flavonoids, carotenoids, alkaloids, and compounds containing nitrogen or organosulfur as potential chemopreventive agents [32]. Accumulating evidence indicates that the development and progression of many cancers, including colorectal cancer, is associated with deregulation of multiple signaling pathways that promote proliferation, inhibit apoptosis and induce metastasis [6]. The Ras and PI3K/AKT pathways have been implicated in the tumorigenesis of colon cancer [6-8,33]. Dietary components such as ginger, garlic, soy, curcumin, chilies and green tea have been shown to reduce the incidence of many cancers [34].

A growing number of *in vitro* and *in vivo* studies have suggested that combinations of dietary phytochemicals targeting multiple signaling pathways are far more effective at enhancing cytotoxic activity and inhibiting the growth of cancer cells [8,9]. In this study, the combination of 3 mg/ml of ginger and 27 mg/ml of Gelam honey was found to be the most effective dose to cause 50% death in HT29 colorectal cancer cells (p<0.05), in contrast to the individual compounds which needed higher concentrations. Isobologram analysis gave a combination index value (CI) of 0.92, indicating a synergistic effect between ginger and Gelam honey as anti-proliferative agents. This dose was found to be equal to the effect of individual Gelam honey treatment at a very high concentration (80 mg/ml) at causing cell death. Similarly, Majumdar *et al*. [35] reported that the combination of curcumin and resveratrol was far more effective at inhibiting colon cancer cells (HCT116) compared to the individual compounds. Furthermore, the combination of two polyphenols, epigallocatechin-3-gallate (EGCG) and curcumin, was found to be more effective than the individual compounds at inhibiting the growth of A549 lung cancer cells and human colorectal cancer cells [36,37].

We found that both individual and combination treatments with ginger and Gelam honey, polyphenol rich foods, induced early apoptosis in HT29 cells in a dose-dependent manner with the combined treatment showing a higher rate of apoptosis. Additionally, ginger alone is far more effective at inducing apoptosis compared to Gelam honey. Abdullah *et al*. [17] showed that ginger oleoresin is capable of inducing early apoptosis and cell cycle arrest in HT29 cells, and Wen *et al*. [24] and Jubri *et al*. [25] showed that Gelam honey induces late apoptosis in HT29 and HepG2 cells, respectively. Nakamura *et al*. [38] reported that the combination of indole-3-carbinol and genistein synergistically induces apoptosis in HT29 cells at a higher rate compared to the single treatments.

To our knowledge, no previous reports have elucidated the signaling pathway mechanisms involved when using the combination of Gelam honey and ginger against the proliferation of HT29 cells. KRAS/ERK and PI3K/AKT are two signaling pathways known to be interconnected and to play a role in colorectal carcinogenesis. The *KRAS*, *ERK* and *AKT* genes have been shown to be critical regulators of colon tumor growth through enhancing survival and reducing apoptosis [6].

We found that both individual and combined treatment with ginger and Gelam honey caused down-regulation of *KRAS* gene expression, which was originally high in the untreated group, but a greater effect was seen in combination treatments using 3 mg/ml ginger with 30 or 50 mg/ml Gelam honey. Budan *et al*. [39] reported that a concoction of tea extracts known as “tea CoDTM”, which contains a combination of Uncaria guianensis, cat's claw (Uncaria sp. U. tomentosa) and Palmer-trumpet tree (Tabebuia sp. T. avellanedae) extracts, inhibits *KRAS* gene expression in the liver, lungs and spleen of DMBA-treated mice.

The present study demonstrated that combining ginger and Gelam honey reduced the expression of the *ERK* and *AKT* genes in HT29 cells more effectively than treatment with the individual compounds. Down-regulation of the *KRAS*, *ERK* and *AKT* genes, which are usually upregulated in proliferating cancer cells, was also observed by Xavier *et al*. [37] who reported that quercetin and luteolin, a type of flavonoid with high antioxidant activity, inhibits growth of HCT115 colon cancer cells by decreasing the gene expression of *KRAS*, *ERK* and *AKT*. Ginger and Gelam honey contain high levels of flavonoids [17,22,24,28], which may be responsible for blocking the activity of *KRAS*, *ERK* and *AKT* and their downstream components.

The PI3K/AKT pathway has been reported to play an important role in cell survival, inhibiting apoptosis through phosphorylation and inactivation of BAD, caspase-9 and forkhead transcription factor family members involved in the intrinsic apoptosis pathway. In addition, it will stimulate the expression of anti-apoptotic proteins such as BCL-2 family, Bcl-xL and MCL-1 [6]. We demonstrated in this study upregulation of the anti-apoptotic gene *Bcl*-*xl* in untreated HT29 cells, but it was downregulated further with combined treatment compared to individual treatment. *Caspase 9* expression increased in a dose-dependent manner with ginger or Gelam honey treatment, but combined treatment showed a greater effect on inducing apoptosis. A similar study by Nakamura *et al*. [38] reported that combination of indole - 3 carbinol with genistein inhibits proliferation of HT29 cells through induction of apoptosis via inhibition of AKT protein phosphorylation and activation of caspase 9 protein, but no effect was seen from single treatment with either indole - 3 carbinol or genistein. The combination of garsinol and curcumin induces apoptosis through activation of caspases 3 and 9 in the PaCa pancreatic cancer cell line [12].

We postulated that the bioactive compounds found in ginger and honey are responsible for inducing apoptosis through activation of apoptosis proteins (BAX, BAD and caspases 3,7, 8 and 9) and inactivation of anti-apoptotic proteins ( BCL-2 and Bcl-xL) as reported by other researchers in KB oral cancer cells, Jurkat T-lymphoma cells, HepG2 cells, p53 hepatoma mutant cells, colon cancer and breast cancer cells [24,40-46].

Activation of nuclear factor kappa B (NFκB) occurs in many diseases including immune diseases, inflammation and cancer [7,20,22,28]. Gelam honey inhibits the production of inflammatory mediators such as NO, PGE2, TNF-α, IL-6, iNOS and COX-2 in carrageenan-induced acute paw edema in rats [28,47]. We observed upregulation and downregulation of *NFκB* (*p65*) and *IκBα* in cancer cells, respectively, which was reversed by treatment with ginger and Gelam honey, with combined treatment showing greater modulation of these genes. Honey showed greater modulation of the genes compared to ginger, while the highest dose of combined treatment (3 mg/ml ginger + 50 mg/ml honey) protected the IκBα protein from destruction, thus preventing the translocation of p65 protein into the nucleus to activate transcription factors involved in cell proliferation and cell survival in HT29 colon cancer cells. Further studies to elucidate fully the mechanism of action of ginger and Gelam honey are required.

## Conclusion

In conclusion, combined treatment with both ginger and Gelam honey is more effective than the individual treatments at inhibiting growth of HT29 colon cancer cells by inducing early apoptosis, modulating the expression of genes involved in the KRAS/ERK/ PI3K/AKT pathways and suppressing inflammation via the NFκB pathway. Thus, the combination of ginger and Gelam honey has potential as a chemopreventive strategy for inhibiting colorectal carcinogenesis by causing cell death.
